# Study of Perfluorophosphonic Acid Surface Modifications on Zinc Oxide Nanoparticles

**DOI:** 10.3390/ma10121363

**Published:** 2017-11-28

**Authors:** Rosalynn Quiñones, Deben Shoup, Grayce Behnke, Cynthia Peck, Sushant Agarwal, Rakesh K. Gupta, Jonathan W. Fagan, Karl T. Mueller, Robbie J. Iuliucci, Qiang Wang

**Affiliations:** 1 Department of Chemistry, Marshall University, Huntington, WV 25755, USA; shoup2@marshall.edu (D.S.); behnke3@marshall.edu (G.B.); peck24@marshall.edu (C.P.); 2 Department of Chemical & Biomedical Engineering, West Virginia University, Morgantown, WV 26506, USA; Sushant.Agarwal@mail.wvu.edu (S.A.); Rakesh.Gupta@mail.wvu.edu (R.K.G.); 3 Department of Chemistry, Pennsylvania State University, State College, PA 16802, USA; jwf188@psu.edu (J.W.F.); Karl.Mueller@pnnl.gov (K.T.M.); 4 Physical and Computational Sciences Directorate, Pacific Northwest National Laboratory, Richland, WA 99352, USA; 5 Chemistry Department, Washington and Jefferson College, Washington, PA 15391, USA; riuliucci@washjeff.edu; 6 Department of Physics and Astronomy, West Virginia University, Morgantown, WV 25606, USA; qiang.wang@mail.wvu.edu; 7 Shared Research Facilities, West Virginia University, Morgantown, WV 25606, USA

**Keywords:** self-assembly films, zinc oxide, perfluorophosphonic acid, solid-state NMR, zeta potential

## Abstract

In this study, perfluorinated phosphonic acid modifications were utilized to modify zinc oxide (ZnO) nanoparticles because they create a more stable surface due to the electronegativity of the perfluoro head group. Specifically, 12-pentafluorophenoxydodecylphosphonic acid, 2,3,4,5,6-pentafluorobenzylphosphonic acid, and (1H,1H,2H,2H-perfluorododecyl)phosphonic acid have been used to form thin films on the nanoparticle surfaces. The modified nanoparticles were then characterized using infrared spectroscopy, X-ray photoelectron spectroscopy, and solid-state nuclear magnetic resonance spectroscopy. Dynamic light scattering and scanning electron microscopy-energy dispersive X-ray spectroscopy were utilized to determine the particle size of the nanoparticles before and after modification, and to analyze the film coverage on the ZnO surfaces, respectively. Zeta potential measurements were obtained to determine the stability of the ZnO nanoparticles. It was shown that the surface charge increased as the alkyl chain length increases. This study shows that modifying the ZnO nanoparticles with perfluorinated groups increases the stability of the phosphonic acids adsorbed on the surfaces. Thermogravimetric analysis was used to distinguish between chemically and physically bound films on the modified nanoparticles. The higher weight loss for 12-pentafluorophenoxydodecylphosphonic acid and (1H,1H,2H,2H-perfluorododecyl)phosphonic acid modifications corresponds to a higher surface concentration of the modifications, and, ideally, higher surface coverage. While previous studies have shown how phosphonic acids interact with the surfaces of ZnO, the aim of this study was to understand how the perfluorinated groups can tune the surface properties of the nanoparticles.

## 1. Introduction

Zinc oxide (ZnO) nanoparticles have distinct properties that allow for a wide variety of applications. For example, it is an n-type semiconducting nanomaterial, which has allowed for its use as a biosensor and as a layer in light emitting diodes [1,2]. Due to its abundance and semiconductive properties, ZnO has been used as an ideal electron transfer layer in inverted solar cells [3,4,5,6,7,8,9]. The implementation of ZnO into electronic and solar devices is largely due to its unique properties, such as a wide band gap (3.37 meV), stable wurtzite crystal structure, and high exciton binding energy (60 meV) [10,11,12,13,14]. Much attention has been focused on modifying the surfaces of ZnO to make it more suitable for applications, such as in heterojunction solar cells and organic light emitting diodes [15,16]. Modifying the surface via organic acid thin films, specifically phosphonic acids, has already shown promise as a means of tailoring and enhancing the aforementioned properties of ZnO [17].

Organic acids are chemically adsorbed onto a surface by forming self-assembled monolayers (SAMs). SAMs have been used to modify surfaces for a variety of purposes, such as forming thiols on the surface of gold for use as a biosensor, modifying metal alloys for use in biomaterials, and serving as a platform for surface initiated polymerization [18,19,20]. Head groups bind to the surface of a substrate via chemisorption, and commonly consist of thiols, phosphonates, and carboxylic acids [21,22,23]. Phosphonic acid SAMs have previously been shown to strongly bind to the surface of zinc oxide and other metal oxides, so they have been utilized to produce favorable changes, such as work function, and hydrophobicity [17,24,25,26]. Interface modifiers with self-assembling properties have been used to improve the charge transfer between organic layers and metal oxides through covalently bonding the modifiers onto the surface of the metal oxide [4]. The modifier can serve multiple purposes, including passivation of the surface charge traps to improve forward charge transfer, tuning of the energy level offset between semiconductors and organic layers, and affecting the upper organic layer morphology [4,27]. The surfaces of the ZnO nanostructures have the potential to be improved via SAMs to make them less corrosive, more stable, and electronically favorable.

There has been interest in using perfluorophosphonic acids to modify ZnO single crystal surfaces in order to fine tune work function and contribute to other favorable characteristics [28]. For example, SAMs of organic phosphonic acids that contain perfluoro groups have been utilized due to the high electronegativity of fluorine and low surface tension, and because phosphonic acids that have tail groups containing fluorine typically have stronger surface adhesion than SAMs with carboxylic acid tail groups [29]. Perfluorophosphonic acids SAMs have also been shown to exhibit a better control of electronic properties than alkyl phosphonic acid SAMs. For instance, they have been used to modify the surface of aluminum and have successfully controlled voltage of thin film transistors [30]. Another ideal property of the perfluorophosphonic acids is that when a fluorinated compound is introduced to a surface, a fluorination effect occurs, which allows for changes in energy levels without steric hindrance [31]. Aromatic perfluorophosphonic acids have previously been shown to have a more densely packed monolayer with a higher fraction of bidentate binding than alkyl chain phosphonic acids, which has contributed to the ability to favorably tune the work function [28,32]. 

**Figure 1:**
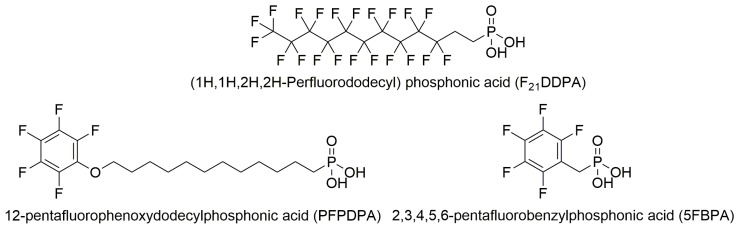
Molecular structures of the perfluorophosphonic acids used to modify the Zinc oxide (ZnO) nanoparticles.

In this study, 12-pentafluorophenoxydodecylphosphonic acid (PFPDPA), 2,3,4,5,6-pentafluorobenzylphosphonic acid (5FBPA), and (1H,1H,2H,2H-Perfluorododecyl)phosphonic acid (F_21_DDPA) were used to form thin films on the surface of ZnO nanoparticles (Figure 1). 

The nanoparticles were characterized using X-ray photoelectron spectroscopy (XPS) (Physical Electronics Inc., Chanhassen, MN, USA) to identify surface composition and surface ratio, and a scanning electron microscope with energy dispersive X-ray spectroscopy (SEM/EDS) (JEOL, Peabody, MA, USA) to observe the morphology and particle sizes of the nanoparticles. Dynamic light scattering (DLS) (Brookhaven Instrument Corporation, Long Island, NY, USA), in addition to the particle size data that is obtained from the SEM images, and zeta potential were used to determine the size and surface charge of the ZnO nanoparticles. Attenuated total reflectance infrared spectroscopy (ATR-IR) (Thermo Fisher Scientific, Waltham, MA, USA) was used to analyze the presence and ordering of the acids that were bonded to the surfaces of the nanoparticles. Solid-state nuclear magnetic resonance (SS-NMR) (Bruker Corporation, Billerica, MA, USA) identified the mode(s) of attachment of the phosphorous on the surface of the ZnO. Thermogravimetric analysis (TGA) (TA Instruments, New Castle, DE, USA) was used to measure the mass changes of the modified samples. 

## 2. Results and Discussion

### 2.1. Attenuated Total Reflectance Infrared Spectroscopy (ATR-IR) 

**Figure 2:**
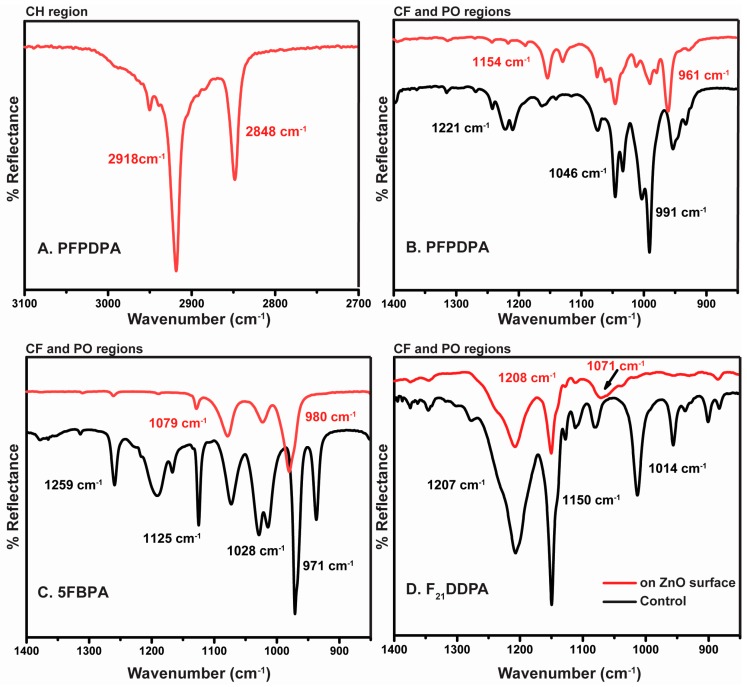
Infrared spectra of ZnO nanoparticles modified with (**A**) PFPDPA C–H region; (**B**) PFPDPA C–F and P–O regions; (**C**) 5FBPA C–F and P–O regions; and (**D**) F_21_DDPA C–F and P–O regions are displayed. The spectral regions occurred after rinsing and sonication (red spectra) compared to each control (black spectra).

ATR–IR was used to distinguish chemisorbed from physisorbed organic thin films on the surfaces of the nanoparticles. In addition, changes on phosphonic head group IR frequencies indicate the manner by which the self-assembled thin films are bonded to the surface. The ZnO nanoparticles were modified via surface chemical adsorption of three organic acids, with phosphonic functional groups (PFPDPA, 5FBPA, and F_21_DDPA). These reactions led to the formation of self-assembled organic thin films on the surface of the nanoparticles. Since ZnO by itself cannot result in detection of organic IR stretches, spectra readily reveal the thin film formation. Rinsing nanoparticles in THF, followed by sonication and vacuum centrifugation is an effective means to remove weakly bound, physisorbed films [19,33,34]. The presence of organic stretches that exist after sonication, as shown in Figure 2, confirm that the attachments were both stable and chemically bonded. 

Typically, the C–H stretches (3000 to 2800 cm^−1^) of SAMs are used as reference bands for SAM organization and the presence of the film on the modified surface [17,35]. However, with the phosphonic acids being used to modify the substrates, only the PFPDPA substrates have these recognizable reference bands, as observed in Figure 2a. There are two characteristic vibrations that are associated with this C–H region: An asymmetrical stretch at ~2918 cm^−1^ and a symmetric stretch at ~2850 cm^−1^. However, these stretches are shifted depending on the conformation of the alkyl chain. Both C–H bands will shift to lower wavenumbers when the alkyl chain is organized on the surface by forming mostly *trans* conformations [36]. After the samples were rinsed and sonicated, the values of $\bar{\nu }$_CH2_ for PFPDPA were 2918 cm^−1^ for $\bar{\nu }$_CH2 asym_ and 2848 cm^−1^ for $\bar{\nu }$_CH2 sym_. The values show that the attachment of the organic acids form strongly-bound and ordered thin films. 

ATR-IR was used to identify the vibrational fingerprints of the molecules that were bonded to ZnO. This allows for the verification of attachment using the P–O vibrations and the analysis of the C–F vibrations to confirm the expected surface functionalization. Adsorbed films exhibit bands in the region from 1400 to 900 cm^−1^ related to C–F and P–O vibrations. In the PFPDPA on ZnO surface IR absorption spectrum (Figure 2b), pronounced bands at 1154, 1046, and 961 cm^−1^ are displayed, which are assigned to the C−F stretching vibration and the P=O and P−O stretching modes of adsorbed phosphonate, respectively. The 5FBPA absorption spectrum (Figure 2c) shows characteristic bands at 1128, 1079, 1023, and 980 cm^−1^, which are assigned to C−F stretching modes of the pentafluorobenzyl group and the formation of phosphonate bonding on the surface. Furthermore, ZnO modified with F_21_DDPA displays fewer bands at 1208, 1150, and 1071 cm^−1^ when compared to the control, as observed in Figure 2d. These bands are assigned to C–F stretching modes of the perfluoroalkyl chain and the formation of a bidentate binding motif for the phosphonate on the ZnO surface, rather than a monodentate or tridentate binding, as previously reported [28,32]. Some reports have claimed that ZnO prefers a bidentate binding motif [17,37]. The relative intensities and placements of the bands (particularly the broad P=O absorption for PFPDPA) and the shift of P=O vibration when compared to the control in the IR spectra suggest mainly bidentate binding of the phosphonic acid for F_21_PPA modifications. Since small signals from free P–O–H vibrations (around 961 and 981 cm^−1^) for 5FBPA and PFPDPA are observed, the possibility that a fraction of the molecules are binding through monodentate attachment and/or a bidentate attachment with a free P–O–H group cannot be ignored.

### 2.2. X-ray Photoelectron Spectroscopy (XPS)

XPS was used to further analyze the bonding motif, the chemical states, and composition of elements of the perfluorophosphonic acid modifications on ZnO nanoparticles. Compositional survey scans were acquired using a pass energy of 117.4 eV and 0.5 eV scan step and high-resolution spectra of Zn2p, O1s, C1s, F1s, and P2p were acquired for both the modified and unmodified ZnO samples using a pass energy of 23.5 eV and 0.05 eV scan step. The representative survey spectrum of unmodified ZnO nanoparticles, presented in Figure S1, in the supporting material, exhibits intense Zn and O peaks, and clearly shows the absence of fluorine and phosphorus, as expected. The insets of Figure S1 show the high-resolution spectra of Zn_2p_ and O_1s_ peaks from the unmodified ZnO control sample.

**Figure 3:**
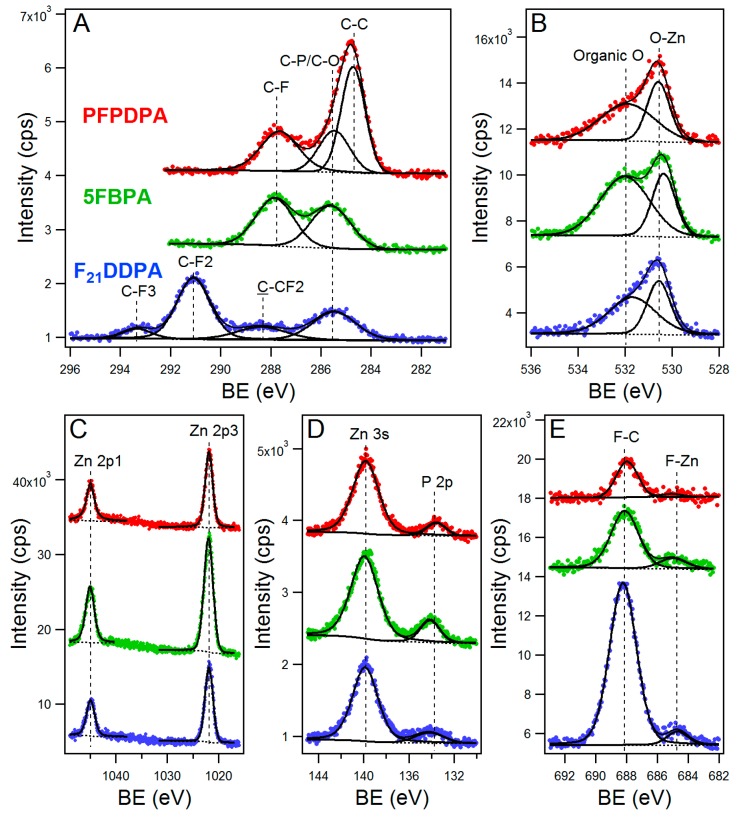
High-resolution X-ray photoelectron spectroscopy (XPS) spectra and fits (**A**) C1s (**B**) O1s (**C**) Zn2p (**D**) P2p, and (**E**) F1s core level spectra of PFPDPA on ZnO (red lines), 5FBPA on ZnO (green lines), and F21DDPA on ZnO nanoparticles (blue lines). Curves are shifted along the Y-axis to enable better comparison.

For the samples that were modified with perfluorinated phosphonic acids, the survey spectra (Figure S1) show the presence of fluorine, carbon, and phosphorus. The high-resolution spectra for each element (Zn, C, O, F, and P) modifications are shown in Figure 3. In addition, Figure 3 shows all of the fitted spectra for each element (Zn, C, O, F, and P) after modification. The binding energy and assigned peaks that were obtained from the fitting are presented in Table S1 in the supporting material. Common to all of the samples, Zn2p at 1022 eV (2p3/2) and 1045 eV (2p1/2) was attributed to the ZnO nanoparticles and O1s at 530.5 eV was attributed to be ZnO. Furthermore, when the samples are modified, new peaks are observed. C1s peaks at 284.8 eV and 287.8 eV were attributed to –CH_2_ and –CF functional groups, respectively. F1s at 688 eV was attributed to C–F functional group. The P2p peak for all of the modifications appears at the similar binding energy around 134 eV, indicating the formation of stable covalent bonds between the oxide surface and the deprotonated phosphonic acid headgroup [38]. As for O1s, a peak at around 532 eV was present and is attributed to oxygen of the phosphonic groups anchored to the ZnO surface [38,39]. When ZnO was modified with F_21_DDPA, two new peaks for C1s at 291 and 293 eV were observed and attributed to –CF_2_–CF_2_ and –CF_2_–CF_3_ groups, respectively [40]. Furthermore, the carbon-to-phosphorus (C/P) and fluorine-to-phosphorus (F/P) ratios were calculated from the relative intensity of the elements and are summarized in Table 1. These numbers are consistent with the molecules that bonded on the surface creating a thin film, and it is a direct measurement of the molecular coverage [24,41]. These values approach the theoretical values shown in Table 1 with the deviation attributed to the possibility of multi-films on the modified surfaces. The agreement between the stoichiometric compositions and the measured C/P and F/P ratios indicate that the films are uniform and homogeneous. Fluorine signal was noticed to increase as the number of fluorine atoms was changed from the PFPDPA and 5FBPA modifications, which had five fluorine atoms, to twenty-one fluorine atoms in the F_21_DDPA modification. This resulted in a corresponding increase in the atomic % concentration and F/P ratios as observed in Table 1. In summary, the XPS results confirm that perfluorophosphonic acid films have modified the surface of the ZnO nanoparticles.

### 2.3. Solid State Nuclear Magnetic Resonance Spectroscopy (SS-NMR)

Solid-state NMR spectroscopy provides a convenient examination of molecular motions and bonding motifs. Here, ^31^P SS-NMR experiments were used to characterize the surface modifications of perfluorophosphonic acid on ZnO nanoparticles.

**Figure 4:**
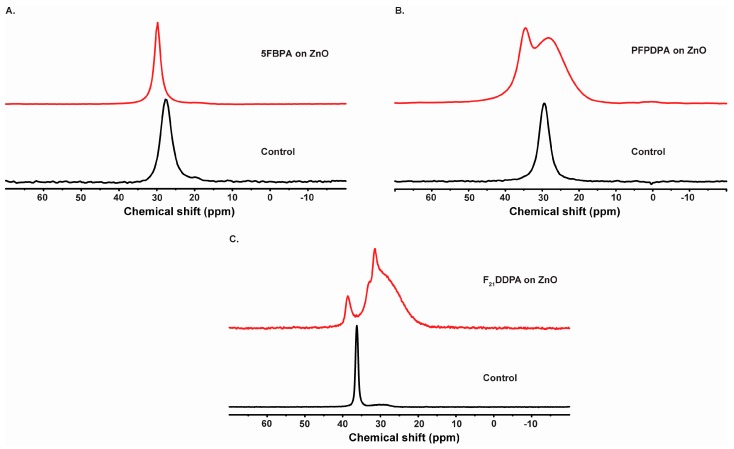
Solid-state ^31^P CP-MAS NMR spectra of (**A**) 5FBPA; (**B**) PFPDPA; and (**C**) F_21_DDPA modifications and bulk controls.

^31^P NMR exhibits only one signal centered at 27.6 ppm for 5FBPA control and at 29.8 ppm for 5FBPA adsorbed on ZnO surfaces, as observed in Figure 4A. In this study, only a single peak is observed, suggesting a preferred hydrogen bonding affinity onto the ZnO surface. This conclusion is due to the fact to the sharpest and least shifted of the peaks is associated with the physisorbed films of the 5FBPA on the ZnO surface, and, therefore, this will not allow for precise conclusion regarding the type of surface bonding [42].

The ZnO samples modified with PFPDPA show two ^31^P resonances between 28.3 and 34.6 ppm, reflecting the option of various bonding motifs while the control has a single peak at 29.4 ppm (Figure 4B). These resonances are broader when compared to the other two modifications and the control, confirming the presence of phosphonate adsorbed to ZnO surface. The peak at 34.6 ppm is likely due to a physisorbed species, while the remaining broad peak arises from a variety of chemisorbed bonding configurations. The broadening effect has been attributed to chemical shift heterogeneity due to a distribution in sites at the nanoparticle interface [43]. Furthermore, ZnO modified with PFPDPA was re-analyzed two years after being modified and the ^31^P SS-NMR spectrum was recorded. This spectrum is shown in Figure 4B and demonstrates that the film remained intact on the surface after ambient storage over a long period of time.

^31^P CP/MAS-NMR spectra of the ZnO modified nanoparticles with F_21_DDPA (Figure 4C) are compared to the spectra of the SS-NMR ^31^P spectra of the bulk F_21_DDPA control. The F_21_DDPA control ^31^P chemical shift changes from a single peak at 36.3 ppm with a small peak at 29.9 ppm. As the ZnO surface was modified with F_21_DDPA, two peaks ranging from 38.6 to 31.5 ppm with the resonance broadened were observed in Figure 4C. The broadening of the peaks was previously noticed due to the possibility of multi-films or multiple bonding sites, as observed by the above XPS data [17,44,45]. Therefore, it is reasonable to associate the three peaks that were observed at lower shift values to the phosphate involved in the surface bonding via monodentate, bidentate, and/or tri-dentate motifs. The peak positioned at 38.6 ppm can be associated to a multilayer stack due to strong hydrogen bonds or fluorine with the –OH groups on the ZnO surface, resulting in some chain torsions and chain motions that may improve the interactions with the ZnO surface [46]. 

### 2.4. Scanning Electron Microscopy-Energy Dispersive X-ray Spectroscopy (SEM/EDS)

**Figure 5:**
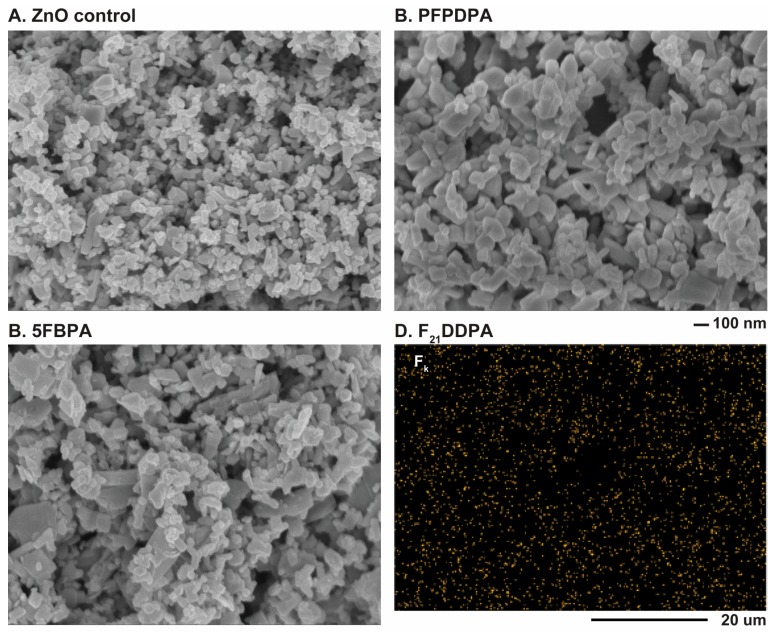
SEM survey spectra of (**A**) ZnO nanoparticles unmodified (control); (**B**) PFPDPA on ZnO; (**C**) 5FBPA on ZnO; and (**D**) energy dispersive X-ray spectroscopy (EDS) fluorine (F_k_) elemental mapping pattern for F_21_DDPA on ZnO nanoparticles.

SEM allowed the visualization of the morphology of the ZnO nanoparticles and EDS assisted in the identification of the elemental composition of the modified ZnO nanoparticles. The SEM images show that both unmodified and modified materials are composed of uniform oblong nanoparticles with typical particle diameters <200 nm. However, a distribution of sizes occurred. There was no visible change in the morphology of the nanoparticles after the modifications of SAMs when compared to the unmodified ZnO nanoparticles (Figure 5). The diameter of the modified nanoparticles is slightly larger than the diameter of the unmodified nanoparticles, which is expected due to the adsorbed perfluorophosphonic acid layer. These measurements are summarized in Table 2. The particle agglomeration that causes the particle sizes to appear to be greater than the value indicated from the manufacturer (>100 nm) is likely due to an increase of interaction between the particles and the charge on the surface, as observed using zeta potential analysis (see below). Aromatic rings are known to have highly delocalized electron density and structural rigidity, and these properties have potential applications in charge transfer and electronic functionality [23,47]. Here, PFPDPA films created more agglomeration when compared to the other two modifications. Nanoparticles that were modified with long alkyl chains, as observed for PFPDPA, have been shown to increase the agglomeration as the order of the films increases [48]. The SEM images confirm that the morphologies of the nanoparticles do not change but the sizes of the particles increase after the surfaces are modified with the phosphonic acids.

EDS elemental analysis obtained in conjunction with the SEM images revealed the presence of zinc, oxygen, fluorine, and phosphorus atoms in all of the examined sections of ZnO modified with 5FBPA, PFPDPA, and F_21_DDPA, whereas the unmodified sample revealed zinc and oxygen atoms. EDS spectra for all of the perfluoro modifications indicates a high relative concentration of fluorine on the surface (Figure S2 in supporting material). Furthermore, EDS mapping (Figure 5D) indicates that fluorine (F_k_) is present homogenously across the sample image, indicating that modification is occurring uniformly, as previously reported [49].

### 2.5. Dynamic Light Scattering (DLS) and Zeta Potential Measurements

DLS was used to measure the particle sizes of the unmodified and modified ZnO nanoparticles in water and THF. The SEM measurements indicate that the particles are significantly smaller (139 to 166 nm) than the results from DLS (217 to 497 nm). DLS measures the hydrodynamic diameter, which is the diameter of the particle and surface associated ligands, ions, or molecules that travel along with the particle in colloidal solution, increasing the average particle size [50]. Therefore, a discrepancy occurs between the two different measurement methods because DLS accounts for this hydrodynamic diameter and Brownian particle displacement of particles in solution, increasing the apparent particle size while SEM does not [48,51]. In SEM, which measures solid surfaces, the counter ion effects and/or electrostatic interactions between the oppositely charged ions are limited when compared to DLS analysis.

Zeta potential is correlated to the surface charge of the particle and the nature and composition of the surrounding medium, in which the particle is dispersed [52]. After surface modification with perfluorinated phosphonic acids, zeta potential values varied significantly. The zeta potential of ZnO was −11.48 mV in THF, and became more negative following modification, with the most negative being −89.12 mV for ZnO nanoparticles that were modified with F_21_DDPA (Table 3). The perfluorinated phosphonic acid modifications were negatively charged, imparting a negative charge to the dispersed nanoparticles, as previously reported [53,54]. This negative charge led to electrostatic repulsion between molecules, stabilizing the nanoparticles. Consequently, the higher absolute value of the zeta potential means an increased stability of the suspended particles against agglomeration [54,55]. All of the perfluorinated phosphonic acid surface modifications that were included in this study led to a significant increase in surface stability when compared to the ZnO control.

Two different solvents with different polarities were used to analyze the stability of the modified surfaces using zeta potential. As shown in Table 3, these observations suggest that the interactions between the ZnO nanoparticles that were modified with perfluorophosphonic acids and the solvent, either water or THF, are relatively significant. While the zeta potential was solvent dependent for the surface modified samples, the zeta potential of unmodified ZnO did not change significantly with a change in solvent. By tailoring the solvent selection process to the desired application, the adsorption capacity of various surface modifications on ZnO nanoparticles could be improved. Additionally, the surface charge is dependent on the solvent viscosity. Usually, viscosity increases as particle size decreases, as shown in Table 3 [56,57]. While water evaporates more slowly than other solvents, allowing for the nanoparticles to remain in solution and preventing the formation of films, which makes it an ideal solvent for nanofluids [57]. THF evaporates more quickly, due to its higher viscosity, and increases the surface stability, as seen in the zeta potential results, making it ideal for use in electronic devices and solar cells applications. Additionally, the surfaces are covered uniformly, as has already been reported for the obtained EDS mapping results. The observed variations in zeta potential may be due to the different solvents used due to their effect on surface chemical composition, surface polarity, and swelling behavior [58]. 

**Figure 6:**
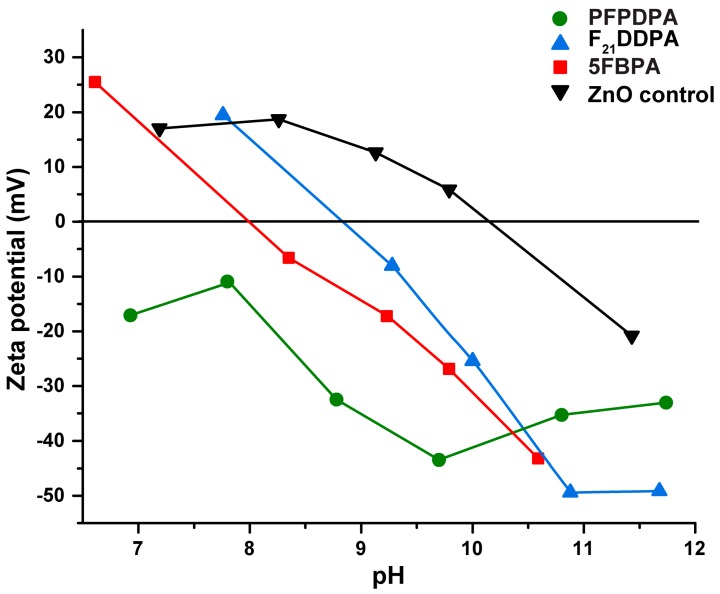
pH dependence of the zeta potentials of surface functionalized ZnO films and unmodified ZnO nanoparticles.

The surface charges of unmodified and modified ZnO nanoparticles were assessed by zeta potential measurements over various pH ranges (Figure 6). Based on prior electrophoresis experiments, it is known that solid oxides in aqueous suspension are generally electrically charged [59]. The isoelectric point (IEP) represents the pH value at which the zeta potential value is equal to zero [60]. Unmodified ZnO nanoparticles reach their isoelectric point at a pH of approximately 10, which agrees with the value that was obtained from a previous study (Figure 6) [61]. The F_21_DDPA and 5FBPA modified ZnO reached their isoelectric point at pHs of 9 and 8.5, respectively. PFPDPA never reaches its isoelectric point and remains negative over the pHs tested. A lack of particle surface charges leads to the absence of inter-particle repulsive forces, causing the colloidal system to be the least stable at the IEP [62]. However, ZnO modified with PFPDPA is not very soluble in aqueous solution, and exhibited a negative surface charge by zeta potential measurements. In neutral solution, ZnO, F_21_DDPA, and 5FBPA are slightly positively charged, and PFPDPA is slightly negatively charged. As previously reported, the zeta potential becomes more negative as the alkyl chain length increases [63]. 

### 2.6. Thermogravimetric Analysis (TGA)

**Figure 7:**
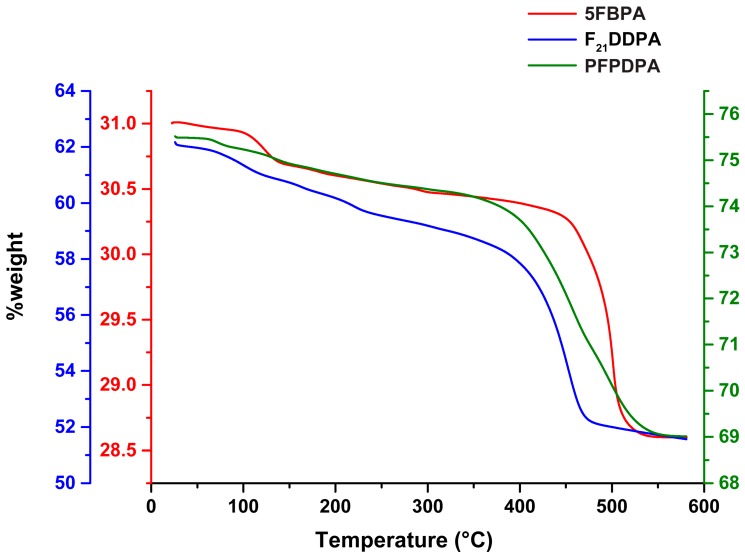
Thermogravimetric analysis (TGA) data analysis of surface modifications with the 5FBPA (red line and *y*-axis), F_21_DDPA (blue line and *y-*axis), and PFPDPA (green line and *y*-axis) modifications on ZnO nanoparticles.

TGA analysis of all the perfluorophosphonic acid modification on ZnO showed a significant weight loss between 350–500 °C, as shown in Figure 7. The overall weight loss is assigned to the decomposition of the surface modifications bonded onto ZnO surfaces [64]. The weight loss in the initial stage is the films that physically absorbed on ZnO surface [65]. The decomposition of 5FBPA had the lowest weight loss % when compared to F_21_DDPA, which has a 14% weight loss, and PFPDPA, which has a 12% weight loss. In addition to a phosphonic acid group, 5FBPA and PFPDPA have a complex benzene ring with resonance possibilities that have C–F functional groups. Therefore, comparing 5FBPA and PFPDPA, PFPDPA has less steric hindrance effect due to the long C–F chain and has well-organized and stronger films on the surface as compared to 5FBPA films [65]. Therefore, 5FBPA has more physically adsorbed films when compared to strongly chemically bonded films for PFPDPA and F_21_DDPA modifications on a ZnO surface. It is concluded that the higher weight loss PFPDPA and F_21_DDPA modifications correspond to a higher surface concentration of the modifications, and, ideally, higher surface coverage [66].

## 3. Materials and Methods

### 3.1. Materials

ZnO nanopowder, PFPDPA, (99.0%), and 5FBPA, (97.0%) were purchased from Sigma Aldrich (St. Louis, MO, USA). F_21_DDPA was purchased from Synquest Laboratories (Alachua, FL, USA). For ZnO nanopowders, the manufacturer reported average particle sizes below 100 nm with a Brunauer-Emmett-Teller surface area of 15 to 25 m^2^/g. Tetrahydrofuran (THF, Optima grade), was purchased from Fisher Scientific (Waltham, MA, USA). All of the chemicals and reagents were used without further purification. 

### 3.2. Preparation of the Samples

For preparation of the adsorbed molecules, 0.35 g of ZnO nanoparticles were dispersed in 30 mL of THF by sonication at 33 ± 2 °C for 15 min. Then, 26.6 mM of each organic acid was added to 6 mL THF and sonicated for 30 min to dissolve. The 30 mL ZnO solutions were combined with the 6 mL acid solutions and sonicated together for 15 min. After sonication, the mixtures were left stirring for 48 h and were allowed to evaporate at room temperature after 24 h. The dry samples were dispersed again in 15 mL of THF and further sonicated for 15 min. The modified nanoparticles were recovered using a vacuum centrifuge (<20 mbar, 1400 rpm for 25 min) and the particles were again left under a fume hood overnight to dry.

### 3.3. Characterization of the Films

#### 3.3.1. ATR–IR

ATR–IR was performed using a Thermo Scientific Nicolet iS50 FT-IR and was used to analyze the alkyl chain ordering and bonding motif of the molecules to the surface. The unmodified ZnO nanoparticles were used to collect a background spectrum for analysis purposes. Typically, 256 scans were collected with a resolution of 2 cm^−1^. 

#### 3.3.2. XPS 

XPS measurements were performed with a PHI 5000 VersaProbe ESCA Microprobe system (ULVAC-PHI). XPS measurements were performed using a focused Al K-Alpha X-ray source at 1486 eV energy and 25 W, with an X-ray spot size of 100 µm. The take-off angle of the photoelectron was set at 45°. An analyzer pass energy of 117.4 eV was used for a survey scan, and high-resolution scans for fluorine, oxygen, phosphorus, and carbon elements were carried out at an analyzer pass energy of 23.5 eV. The XPS spectra were referenced to the C1s peak at a binding energy of 284.8 eV. 

#### 3.3.3. SS-NMR 

Solid state NMR spectra were acquired with a Bruker Avance 300 spectrometer and 7 T Bruker magnet. Samples were packed into 4 mm zirconia rotors. A Bruker double resonance MAS probe was tuned to a ^1^H frequency of 300.405 MHz and a ^31^P frequency of 121.606 MHz. The ^31^P direct-polarization pulse sequence used a 3.50 μs 90° pulse on the ^31^P channel and 30 kHz of proton decoupling during acquisition. The free induction decay (FID) was Fourier transformed with 16 Hz of line broadening after zero filling to a total of 32 k points. The ^31^P spectra were referenced externally by measuring the resonance frequency of an aqueous solution of 85% phosphoric acid (set to 0.0 ppm).

#### 3.3.4. SEM/EDS 

SEM/EDS was performed using a JEOL JSM-7600F field emission SEM. The EDS was collected with an Oxford INCA EDS system and data were analyzed using the Oxford Aztec Energy Analyzer software. The chamber of the SEM was held under high vacuum conditions. The accelerating voltage for the EDS ranged from 3–10 kV. Samples were prepared individually in pin stubs and were sputtered with a 10-nm thin coat of gold/palladium. SEM/EDS was used to analyze the surface composition of the nanoparticles and obtain information about particle size and elemental composition. 

#### 3.3.5. DLS/Zeta Potential

A Brookhaven ZetaPlus Potential Analyzer (90Plus PALS) was used to perform DLS and zeta potential measurements of the unmodified and modified ZnO nanoparticles. The measurements were performed at 25 °C in water and THF. At least three measurements were made for each sample, and the collected values were averaged. The Zeta Potential Analyzer was employed to determine the direction of particles under the influence of an electric field, allowing for the estimation of the zeta potentials of the nanoparticles.

#### 3.3.6. TGA

The thermogravimetric analysis was acquired with a TA Q500 TGA at a heating rate of 10 °C/min and a flow rate of high purity nitrogen of 100 mL/min. Approximately 20–30 mg of samples was measured in alumina pans.

## 4. Conclusions

In this study, ZnO nanoparticles were modified by self-assembly of perfluorinated phosphonic acids. The IR and XPS data showed that the modifications attached at the phosphonic head group and formed strong and ordered thin films on the surfaces of the nanoparticles. The IR and SS-NMR spectra also indicated that these films remained strongly bonded on the surfaces after sonication and after two years. 

SEM images indicate that the morphologies of the nanoparticles do not change with the modifications from their initial oblong shape, and EDS mapping data shows that the modifications are homogenously distributed throughout the samples. As expected, particle sizes from SEM indicate that the sizes of the nanoparticles increase with the addition of the perfluorinated phosphonic acids. However, DLS particle sizing shows a decrease in particle sizes after modification, which correlates to the zeta potential measurements that show the surface stability of the modified nanoparticles increases when compared to the unmodified ZnO. Over a range of pH values, the ZnO control IEP corresponds with literature values, while the modified samples IEP decreased (5FBPA and F_21_DDPA) or remained negative, and never reached an isoelectric point (PFPDPA modification). Thermogravimetric analysis indicated that higher weight loss corresponds to less steric hindrance and chemically bonded films, rather than physically bonded films. 

These results indicate that modifying the ZnO nanoparticles with perfluorinated phosphonic acids increases the stability of the phosphonic acids that are adsorbed on the surfaces, as revealed by zeta potential measurements, even though multi-layer or physisorbed films (as shown by XPS and SS-NMR analysis) are formed that show sufficient stability. Here, both XPS and SS-NMR analysis indicate a heterogeneous population on the ZnO surface, involving various bonding motifs that are presumably bound at different types of adsorption sites. While it is already understood how phosphonic acids and the surfaces of ZnO interact, this study is a crucial step in understanding how perfluorinated groups can tune the surface properties of the nanoparticles. These modified nanoparticles could be incorporated into systems where a stable surface is necessary.

## Figures and Tables

**Table 1:** Atomic percentages and C/P and F/P ratios determined by XPS and compared with theoretical values calculated from the chemical formula (numbers in parenthesis).

Sample	Zn2p %	O1s %	C1s %	F1s %	P2p %	C/P	F/P
**PFPDPA**	14.9	28.3	44.9	10.1	1.8	24.8 (18)	5.5 (5)
**5FBPA**	25.0	31.2	23.5	17.6	2.7	8.7 (7)	6.5 (5)
**F_21_DDPA**	13.5	19.5	25.9	39.1	2.1	12.4 (12)	18.7 (21)
**ZnO Control**	47.5	52.5	-	-	-	-	-

**Table 2:** Particle size and distribution obtained from scanning electron microscope (SEM).

Modifications	Average Particle Size (nm)	Particle Distribution (±nm)
ZnO	139.8	18.6
5FBPA	167.0	27.9
PFPDPA	194.1	40.8
F_21_DDPA	166.0	32.7

**Table 3:** Particle size and zeta potential values of the hydrodynamic diameters of ZnO and surface modified nanoparticles obtained using dynamic light scattering (DLS).

Modification	Water		THF	
	Particle Size (nm)	Zeta Potential (mV)	Particle Size (nm)	Zeta Potential (mV)
ZnO	497.2 ± 12.2	−11.09 ± 0.42	413.2 ± 18.0	−11.48 ± 4.40
5FBPA	247.1 ± 8.9	−20.31 ± 0.66	279.1 ± 3.5	−48.04 ± 2.68
PFPDPA	275.3 ± 9.5	−22.53 ± 0.39	243.5 ± 0.7	−52.29 ± 3.02
F_21_DDPA	217.5 ± 8.4	−18.64 ± 0.56	244.1 ± 2.2	−89.12 ± 1.96

## Supplementary Materials

The following are available online at www.mdpi.com/1996-1944/10/12/1363/s1, Table S1: Binding energies determined by XPS, Figure S1: XPS survey spectra, Figure S2: EDS spectra for modified ZnO nanoparticles.
