# Supplementary material for: Study of Perfluorophosphonic Acid Surface Modifications on Zinc Oxide Nanoparticles
Source: Materials (Basel). 2017 Nov 28;10(12):1363. doi: 10.3390/ma10121363 (PMC5744298; doi:10.3390/ma10121363)
Supplement: Supplementary file 1 [file materials-10-01363-s001.pdf]

materials

Supplementary Material

Study of Perfluorophosphonic Acid Surface Modifications on Zinc Oxide Nanoparticles

Rosalynn Quiñones*[1], Deben Shoup[1], Grayce Behnke[1], Cynthia Peck[1], Sushant Agarwal[2], Rakesh K. Gupta[2], Jonathan W. Fagan[3], Karl T. Mueller[3,4], Robbie J. Iuliucci[5], and Qiang Wang[6,7]

[1]Department of Chemistry, Marshall University, Huntington, WV 25755, USA
[2]Department of Chemical & Biomedical Engineering, West Virginia University, Morgantown, WV 26506, USA
[3]Department of Chemistry, Pennsylvania State University, State College, PA 16802, USA
[4]Physical and Computational Sciences Directorate, Pacific Northwest National Laboratory, Richland, WA 99352, USA
[5] Chemistry Department, Washington and Jefferson College, Washington, PA 15391, USA
[6]Department of Physics and Astronomy, West Virginia University, Morgantown, WV 25606, USA
[7]Shared Research Facilities, West Virginia University, Morgantown, WV 25606, USA
*Corresponding author email: quinonesr@marshall.edu; Phone: 1+ (304) 696-6731, Fax: 1+ (304) 696-3243

Received: 27 October 2017; Accepted: 22 November 2017; Published: 28 November 2017

Table S1. Binding energies determined by XPS.

	ZnO control	PFPDPA	5FBPA	F_{21}DDPA
	Binding energy (eV)	Binding energy (eV)	Binding energy (eV)	Binding energy (eV)
**Zn2p3/2**	1022.0	1021.9	1021.9	1021.9
**Zn2p1/2**	1045.1	1045.0	1045.0	1044.9
**O1s**	530.6	530.6	530.4	530.6
	531.8	531.9	532.0	531.7
**C1s**		284.8	287.9	293.3
		285.5	285.6	291.1
		287.7		288.4
				285.5
**P2p**		133.6	134.1	134.1
**F1s**		688.0	688.1	688.2
		685.0	685.0	684.7

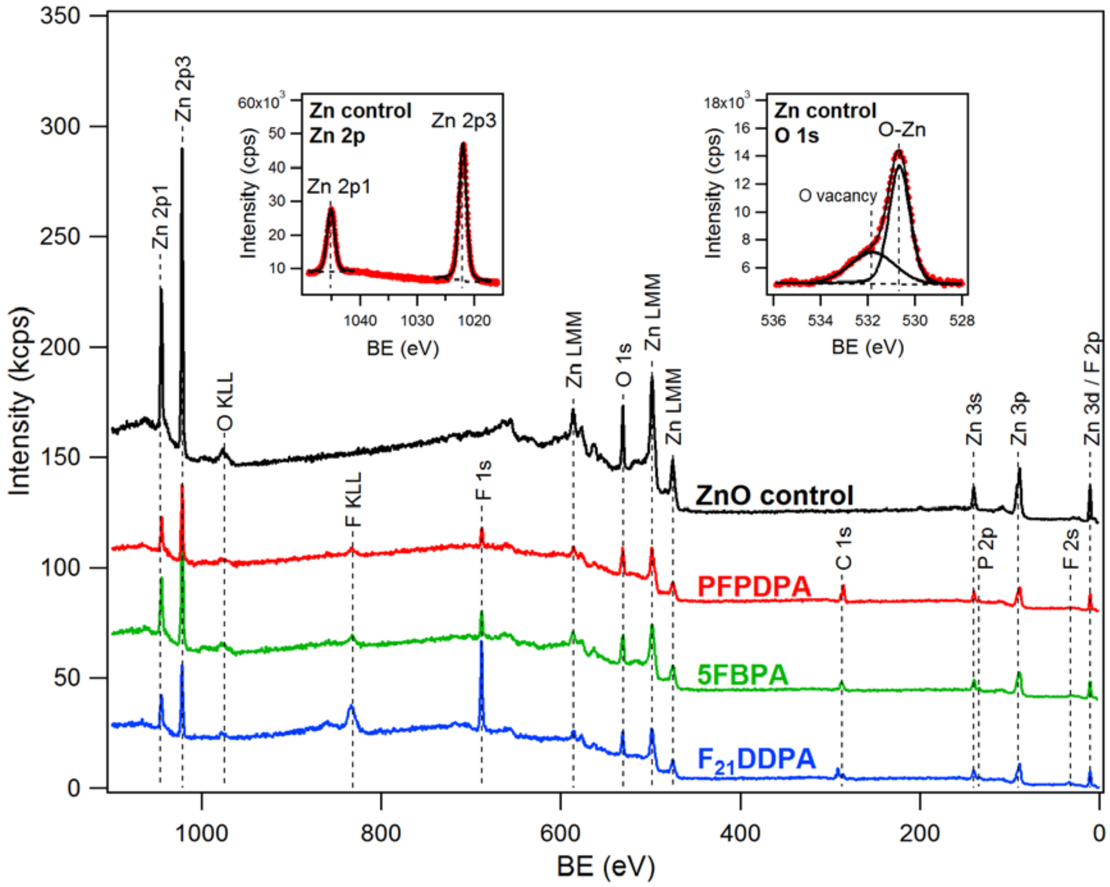

Figure S1. XPS survey spectra of (a) ZnO nanoparticles unmodified (control) (black line), (b) PFPDPA on ZnO (red line), (c) 5FBPA on ZnO (green line), and (d) F_{21}DDPA on ZnO nanoparticles (blue line). High-resolution core level spectra of ZnO unmodified (black) for Zn2p and O1s are also shown.

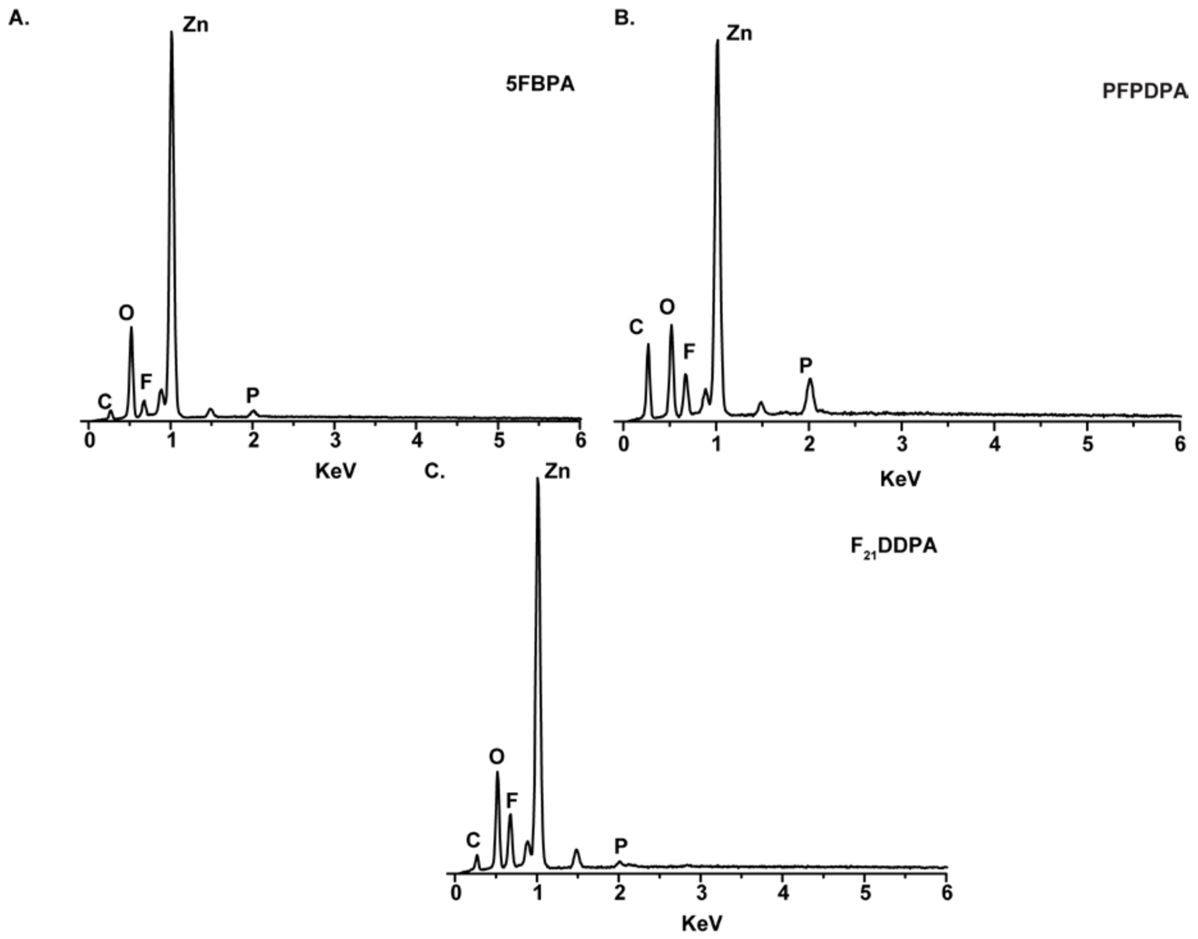

Figure S2. EDS spectra for (a) 5FBPA, (b) PFPDPA, and (c) F_{21}DDPA modified ZnO nanoparticles.
